# Sinogram Interpolation Inspired by Single-Image Super Resolution

**Published:** 2023-05-15

**Authors:** Carolyn Christiansen, Gengsheng L. Zeng

**Affiliations:** 1 Department of Computer Science, Utah Valley University, Orem, Utah, USA.; 2 Department of Radiology and Imaging Sciences, University of Utah, Salt Lake City, Utah, USA.

**Keywords:** Deep learning, Limited data imaging, Machine learning, Medical imaging, Tomography

## Abstract

Computed tomography is a medical imaging procedure used to estimate the interior of a patient or an object. Radiation scans are taken at regularly spaced angles around the object, forming a sinogram. This sinogram is then reconstructed into an image representing the contents of the object. This results in a fair amount of radiation exposure for the patient, which increases the risk of cancer. Less radiation and fewer views, however, leads to inferior image reconstruction. To solve this sparse-view problem, a deep-learning model is created that takes as input a sparse sinogram and outputs a sinogram with interpolated data for additional views. The architecture of this model is based on the super-resolution convolutional neural network. The reconstruction of model-interpolated sinograms has less mean-squared error than the reconstruction of the sparse sinogram. It also has less mean-squared error than a reconstruction of a sinogram interpolated using the popular bilinear image-resizing algorithm. This model can be easily adapted to different image sizes, and its simplicity translates into efficiency in both time and memory requirements.

## Introduction

In medical imaging, any patient exposure to radiation means risk. Hence, it is ideal and morally sound to limit this exposure. However, this limitation in exposure translates to a sparsity of data for medical scans, and potential loss in imaging information. The goal of this project is to construct a reasonably detailed representation of imaged objects using sparse data.

Computed tomography (CT) is a medical imaging procedure used to estimate the interior of a patient or an object. Radiation scans are taken at regularly spaced angles around the object, forming a sinogram. This sinogram is then reconstructed into an image representing the contents of the object [1]. This results in a fair amount of radiation exposure for the patient, which increases the risk of cancer. A successful model means less radiation can be used in a scan. Better images provide better information for doctors, which can produce better decisions. This will hopefully translate into better patient outcomes.

More views mean more radiation exposure for the patient. However, using current methods, the under-sampling leads to artifacts and inferior image reconstruction. Several methods of solving this sparse-view problem have been proposed. Algorithms operate in various domains in this endeavor (reconstructed image domain or sinogram domain). Many, but not all, of the solutions involve deep learning.

Some methods remove artifacts in the reconstructed image. Outside of machine learning, an iterative algorithm [2] has been used. Various deep learning models applied to this problem include a general adversarial network (GAN) [3], U-net model [4], and a model employing wavelet transform [5]. Other methods attempt to interpolate the sinogram. Outside of machine learning, a sine wave approximation technique [6] has been proposed. Deep learning models applied to this problem include a U-net model [7], and a U-net and residual model [8]. Yet other deep learning models work in both domains [5,9,10]. These complex methods attempt to combine information from the sparse sinogram and the reconstructed image.

The model presented here falls into the second category, interpolating the sparse sinogram and reconstructing the interpolation. As the model will be based on SISR, several deep learning models that have been applied to generic image reconstruction should be noted. A few of the current models are the Super-Resolution Convolutional Neural Network (SRCNN) [11], residual models including MSRN [12,13] and EDSR [14], and the inception model [15]. There are other ways to extend the sparse data such analytic extension [22,23], deformation [24], nonlinear filtering [25], and so on.

The model attempts to solve this sparse-view problem by interpolating the sinogram using techniques from single image super-resolution (SISR). In the literature review, no papers were found that use this exact method to estimate a full sinogram from a sparse sinogram. Specifically for parallel-beam CT, the deep learning model will accept the input of a sparsely sampled sinogram and output the prediction for a corresponding full sinogram. The goal of the model is that predicted full sinogram should be a reasonable reconstruction of the original image, with the error less than that of a reconstruction of the sparse sinogram.

## Methods

### Phantoms

In developing and testing medical technology, simulated images called *phantoms* are used instead of real patients (or patient data). This practice is common in the field, as it provides simulated data that can be used to test a model.

In the first stage of the project, phantoms of one or two ellipses of varying intensities are generated on a black background [16]. Anything outside the reconstruction circle is blacked out to allow direct comparison of the phantom with the model predicted reconstruction. These represent ground truth, or the ideal expected result. There are 1000 images of size 128 × 128 area generated. These are used to train the model. There are 250 additional images that are generated to test the model.

**Figure 1:**
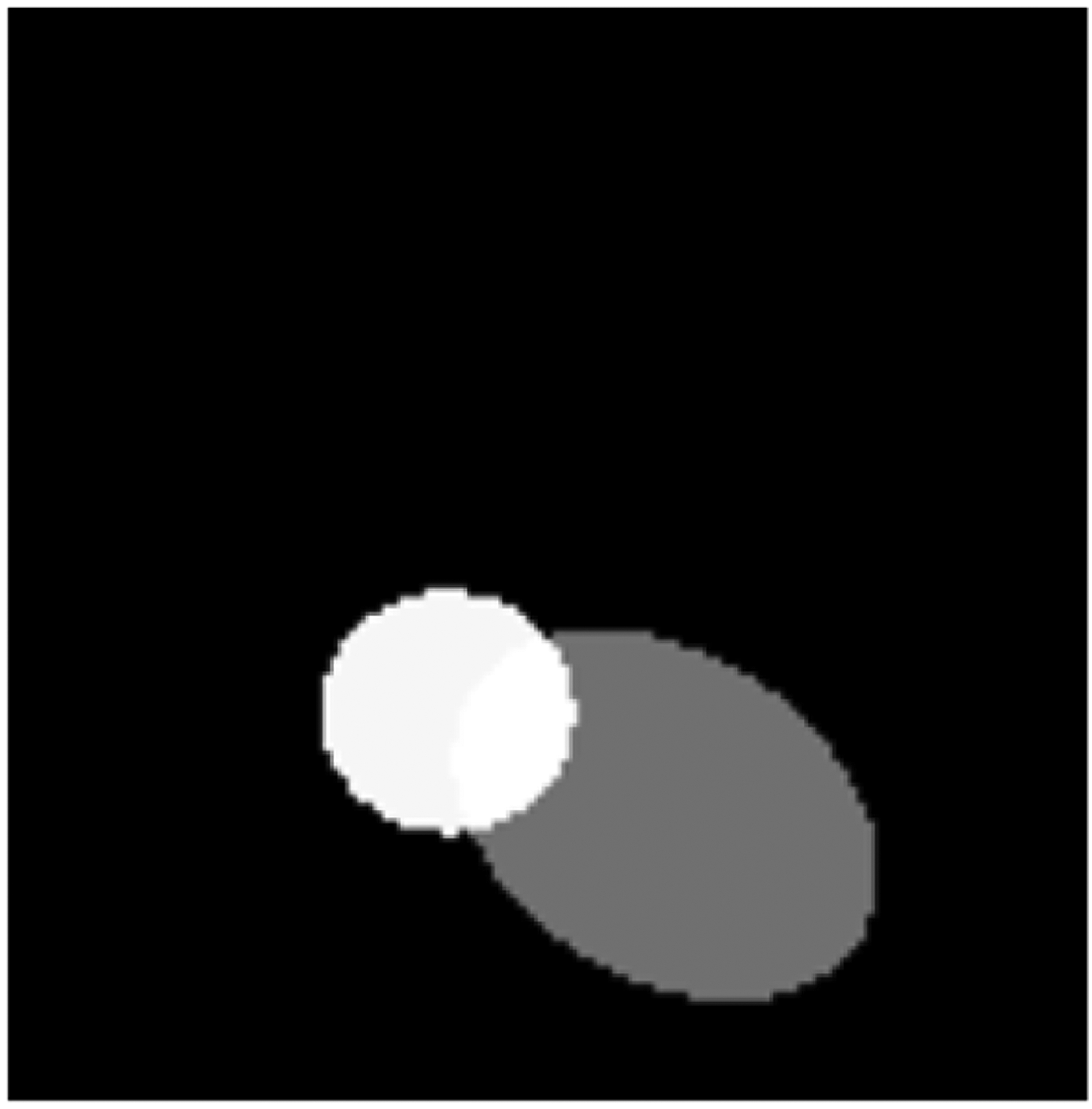
A simple elliptical phantom.

**Figure 2:**
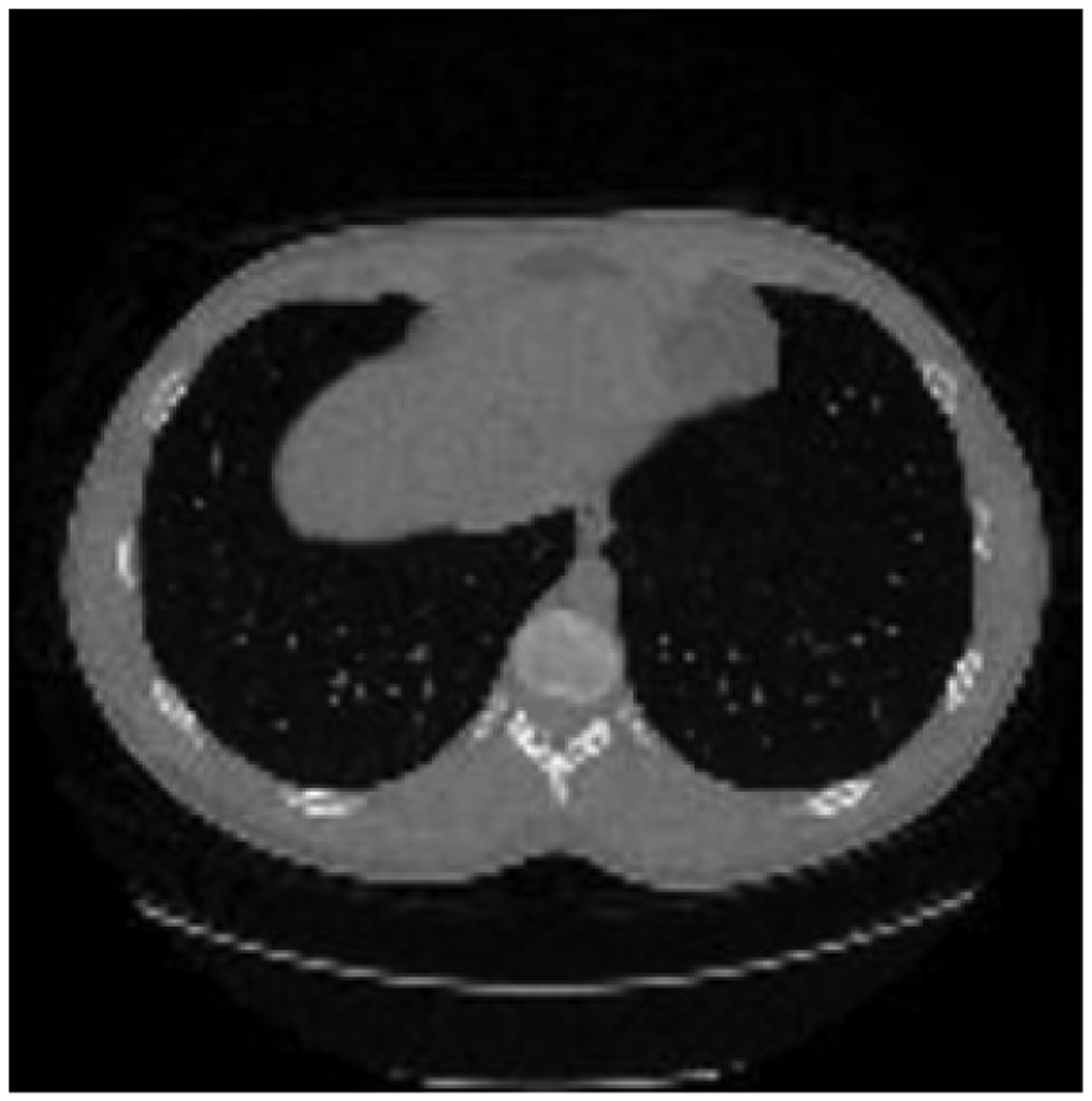
A CT image from TCIA dataset.

In the second stage of the project, clinical reconstructed CT images are downsized and used as more complex, realistic phantoms. Clinical images are obtained from The Cancer Imaging Archive [17–19] and are all of size 512 × 512 pixels. The 1192 images are randomly chosen from 298 patients and resized to 128 × 128 pixels to use as phantoms to train the model. Another 298 are randomly chosen to test the model. The sets of test images and training images are disjoint. Sample phantoms are shown in Figures 1 and 2.

### Sinograms and Ct Image Reconstruction

Computed tomography (CT) is a medical imaging procedure used to estimate the interior of a patient or an object. For parallel-beam CT, one-pixel-tall radiation scans are taken at regularly spaced angles around the object (Figure 2) and then stacked on top of each other, forming a sinogram (Figures 13–16). Applying the Central Slice Theorem and a process called filtered backprojection (FBP), this sinogram is then reconstructed into a two-dimensional image representing a slice of the contents of the object, as shown in Figures 9–12 and 17–20 [1]. Several slices are usually taken in the same patient.

It should be noted that the number of views in a true, “gold-standard” sinogram will be much greater in clinical practice. These numbers are chosen for ease in viewing artifacts. According to Zeng [20], for a scan using 896 detectors, measurements from 1200 views over 360° are considered as a full sinogram, and measurements from 400 views over 360° are considered as an under-sampled sinogram.

In sparse-view tomography, measurements are under-sampled, which usually result in severe aliasing artifacts as streaking lines in the reconstructed images. Hence, a sparse sinogram will contain 1/3 the views of a full sinogram, or fewer. For the image in Figure 2, a sparse sinogram with 16 views is represented by Figure 13, and artifacts are obvious in the reconstruction (Figure 17). Success of the project is measured by how closely the model-predicted reconstruction matches the original phantom, which is ground truth. In a clinical setting, however, this ground truth is impossible to access, since an accurate picture of the patient’s interior is unavailable. Hence, any reconstructed image will be compared to the full-sinogram reconstruction using the mean-squared error metric. The model cannot be reasonably expected to perform better than the reconstruction of the full sinogram, which is the model’s target. However, the goal is to produce results superior to the image reconstructed from the sparse sinogram.

### Image Resizing Algorithms

Several algorithms exist for resizing an image. The bilinear algorithm (called *linear* throughout the paper) is one of these. It uses linear approximation in two dimensions to approximate pixel values when resizing an image. The model does not use this method, but it provides a useful comparison to show the deficiencies of a different algorithm used to solve the same problem to interpolate a full sinogram from a sparse one.

### Single Image Super-Resolution

**Figure 3:**
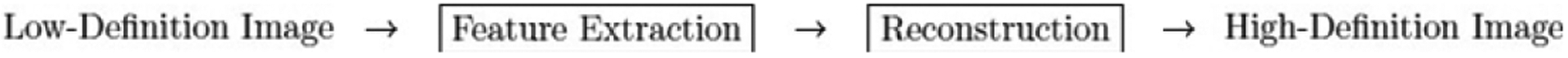
Common architecture for SISR.

**Figure 4:**
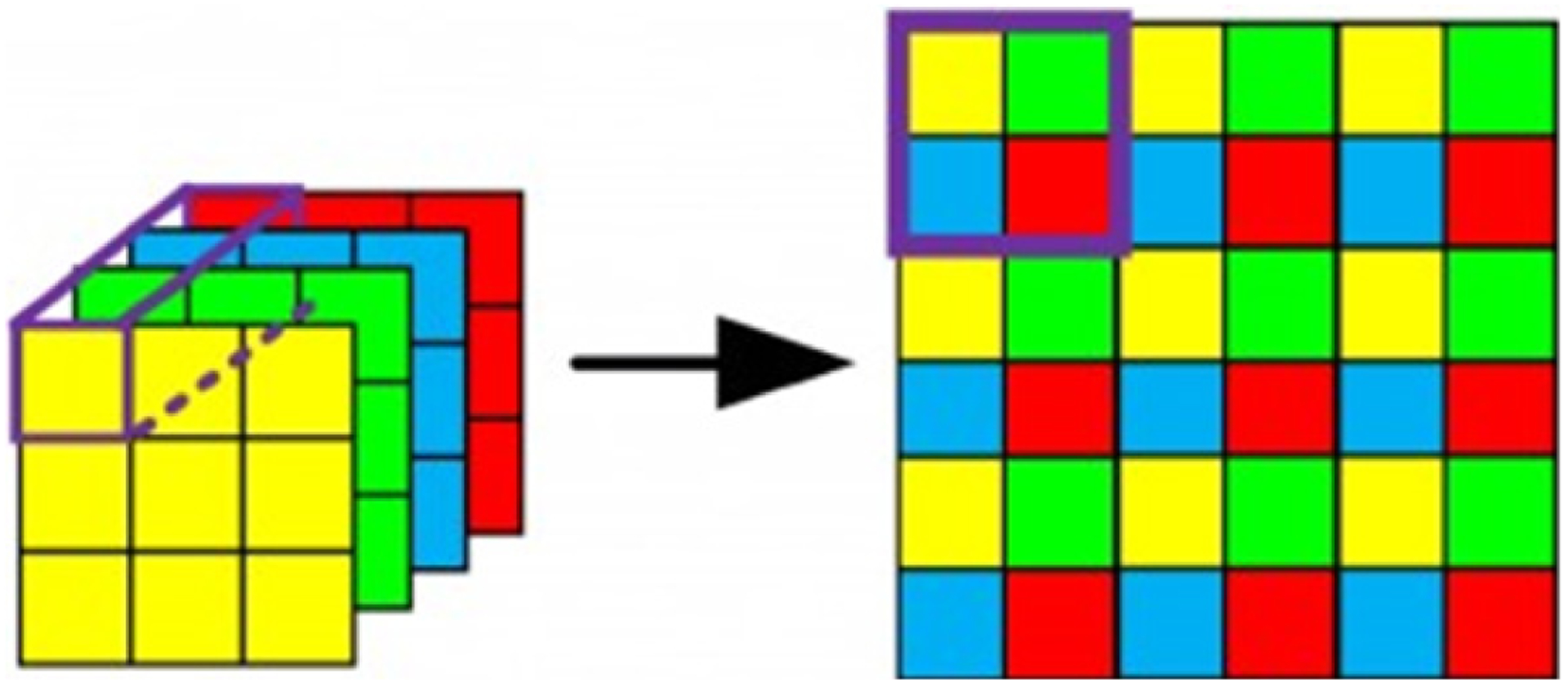
Visual representation of pixelshuffle.

Single image super-resolution is a process that approximates high-definition images from low-definition images. Most models for SISR have two main parts (shown in Figure 3): feature extraction of the low- definition image, and reconstruction of a high-definition object using a transposition layer (sometimes called a pixel shuffle layer). This layer converts several feature layers into one larger layer by rearranging the tensor (pixel) values. To illustrate, Figure 4 represents pixel shuffle from four layers to one layer.

### Deep-Learning Model Architecture

**Figure 5:**
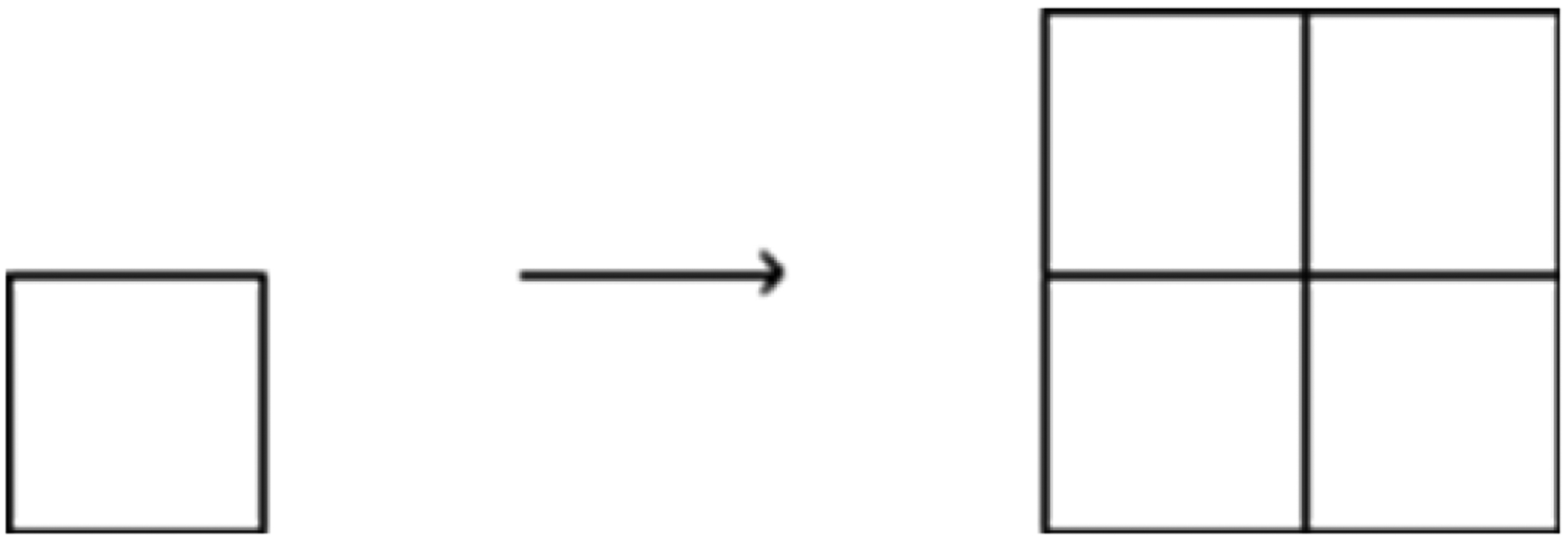
Classic SISR: Each pixel of the input image is extended in both dimensions.

**Figure 6:**
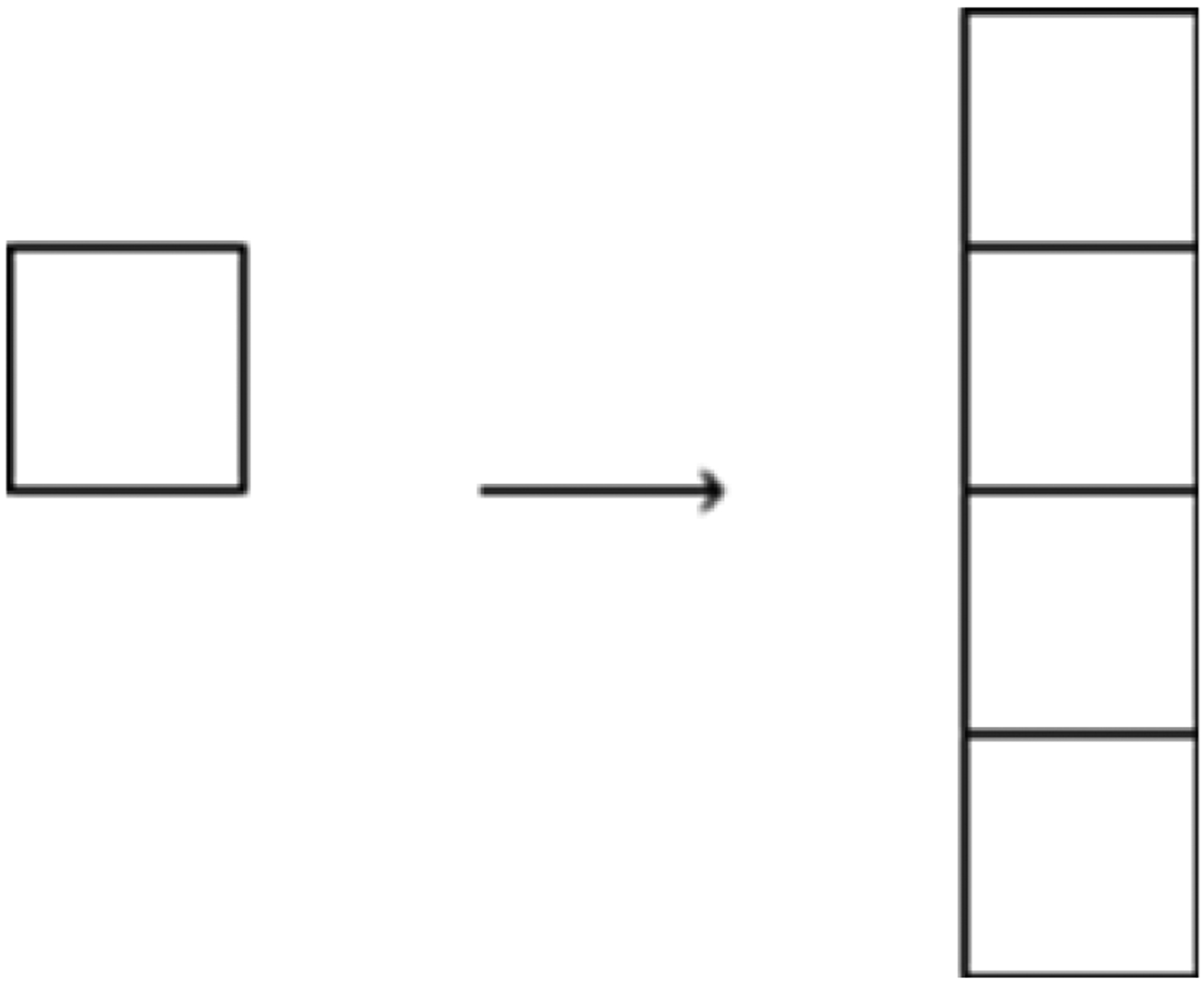
Sinogram interpolation: The sinogram is only extended in the angular (vertical) dimension.

The sparse sinograms contain 1/4 of the views of the full sinograms. The project goal is to approximate the full sinograms from the sparse sinograms. Sinogram approximation closely resembles the goal of single-image super resolution (SISR). SISR aims to grow the image in both dimensions, maintaining the aspect ratio of the low-definition image (Figure 5). This sinogram approximation aims only to expand the image in one dimension. In this particular case, it must grow four times in the angular (vertical) direction (Figure 6).

TensorFlow is a software library that focuses on training deep neural networks. Using the Python application-programming interface Keras to access TensorFlow functionality, a deep-learning model is constructed. As inputs to the model, “sparse” sinograms with 16 views are created by applying Python’s radon() function to the training images (Figure 13). As targets of the model, “full” sinograms with 64 views are created by applying Python’s radon() function to the training phantoms (Figure 16).

**Figure 7:**
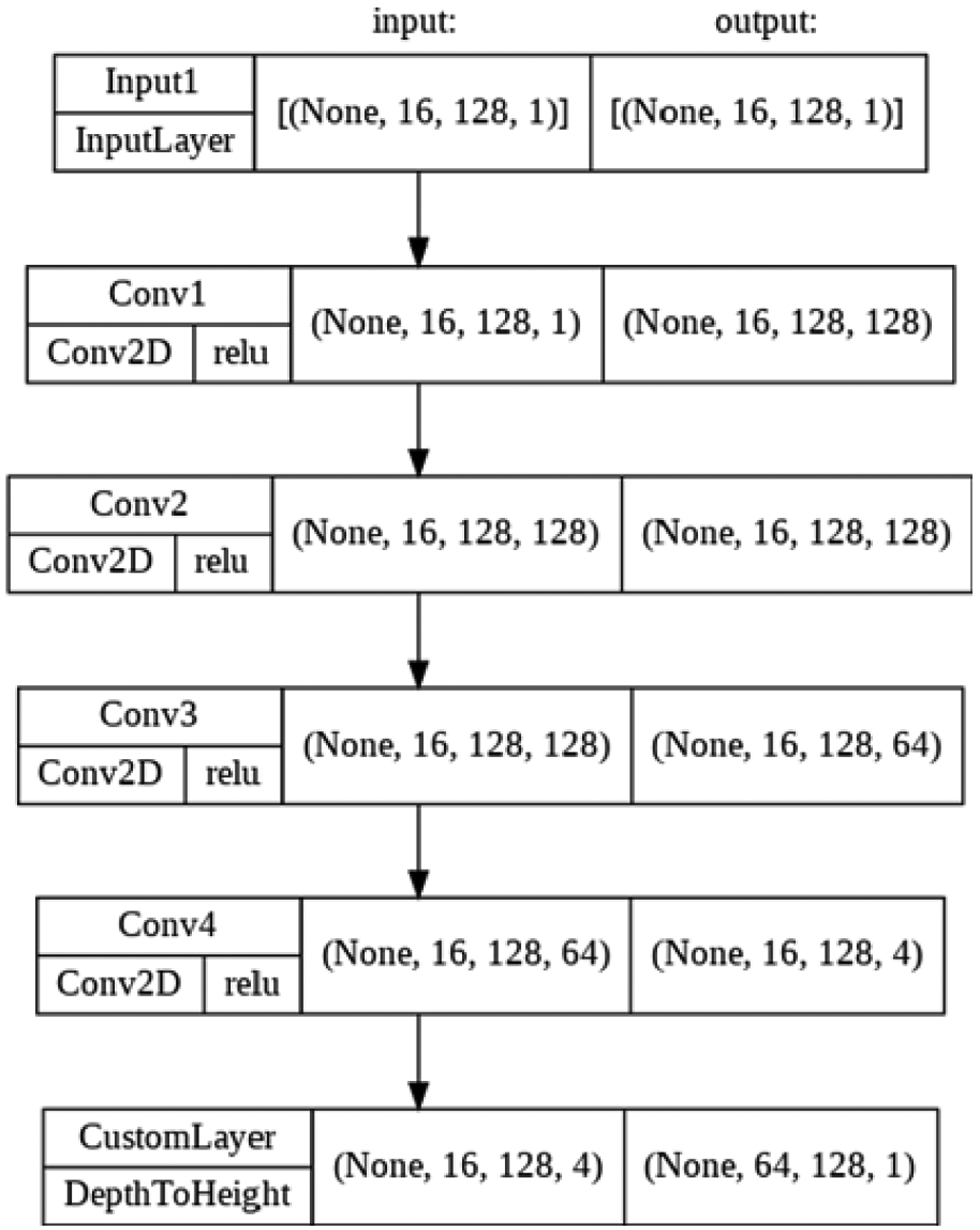
Model architecture.

**Figure 8:**
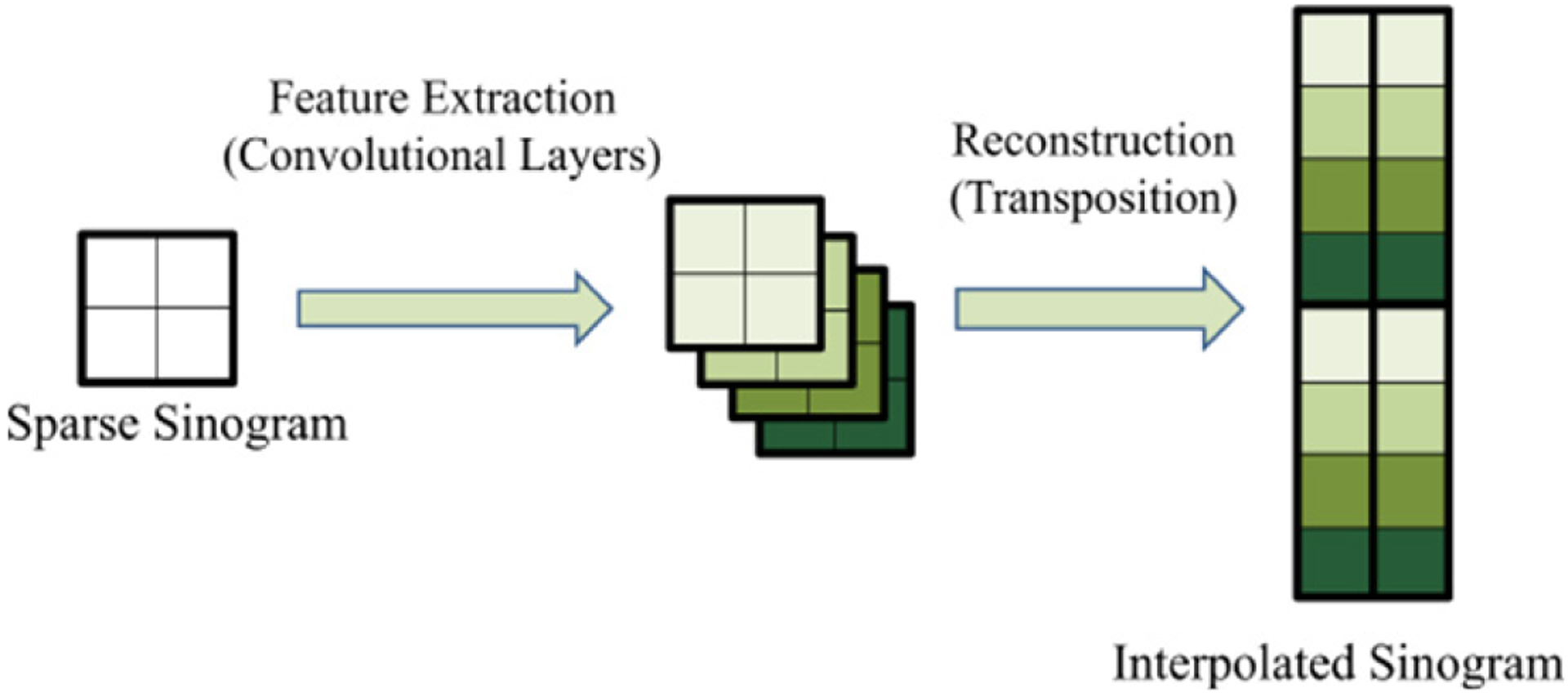
Model architecture demonstrating custom transposition layer.

After trying many model architectures for infilling sparse sinograms, the best results have come from a few simple dense 2D Convolutional layers (a super-resolution convolutional neural network [11]) followed by a modified pixelshuffle tail, as shown in Figure 7. All kernel sizes are 3 × 3. This modified pixelshuffle tail is a custom transposition layer. At the end of feature extraction, each pixel of the input image corresponds to four pixels, one on each feature layer. These four pixels must be transposed onto one layer, stacked vertically, to form the output-interpolated sinogram (Figure 8).

The random generation of initial model weights introduces uncertainties into this process. Another experimenter using the same code and data may produce a slightly different model.

### Model Advantages

This model is simple, as compared to many SISR models. This translates into efficiency in time and memory space. From the author’s experience, this model is generally faster than a residual model. It also may occupy less space in memory than a model that upsizes the sparse sinogram before applying filters and other feature extraction strategies.

A strong advantage of this model is the flexibility of input and output sizes. The input sparse sinogram may be any size, and the interpolated output full sinogram may be any size that is a multiple of the input size. Dimensions are not constrained to powers of 2, as in some u-net models, and the input sinogram does not need to be resized before the model can accept it.

## Results

**Figure 9:**
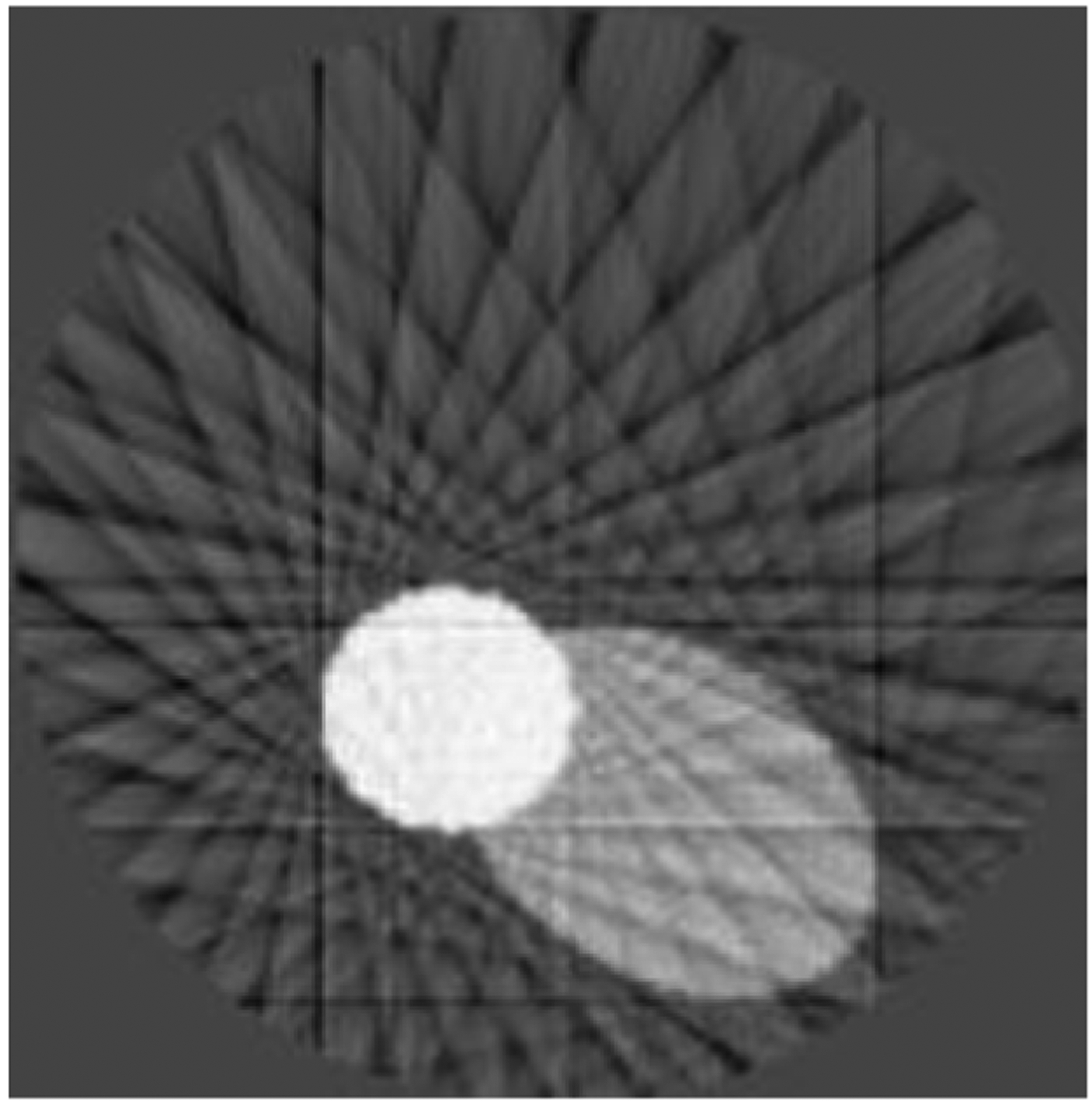
Reconstruction from sparse sinogram of elliptical phantom.

**Figure 10:**
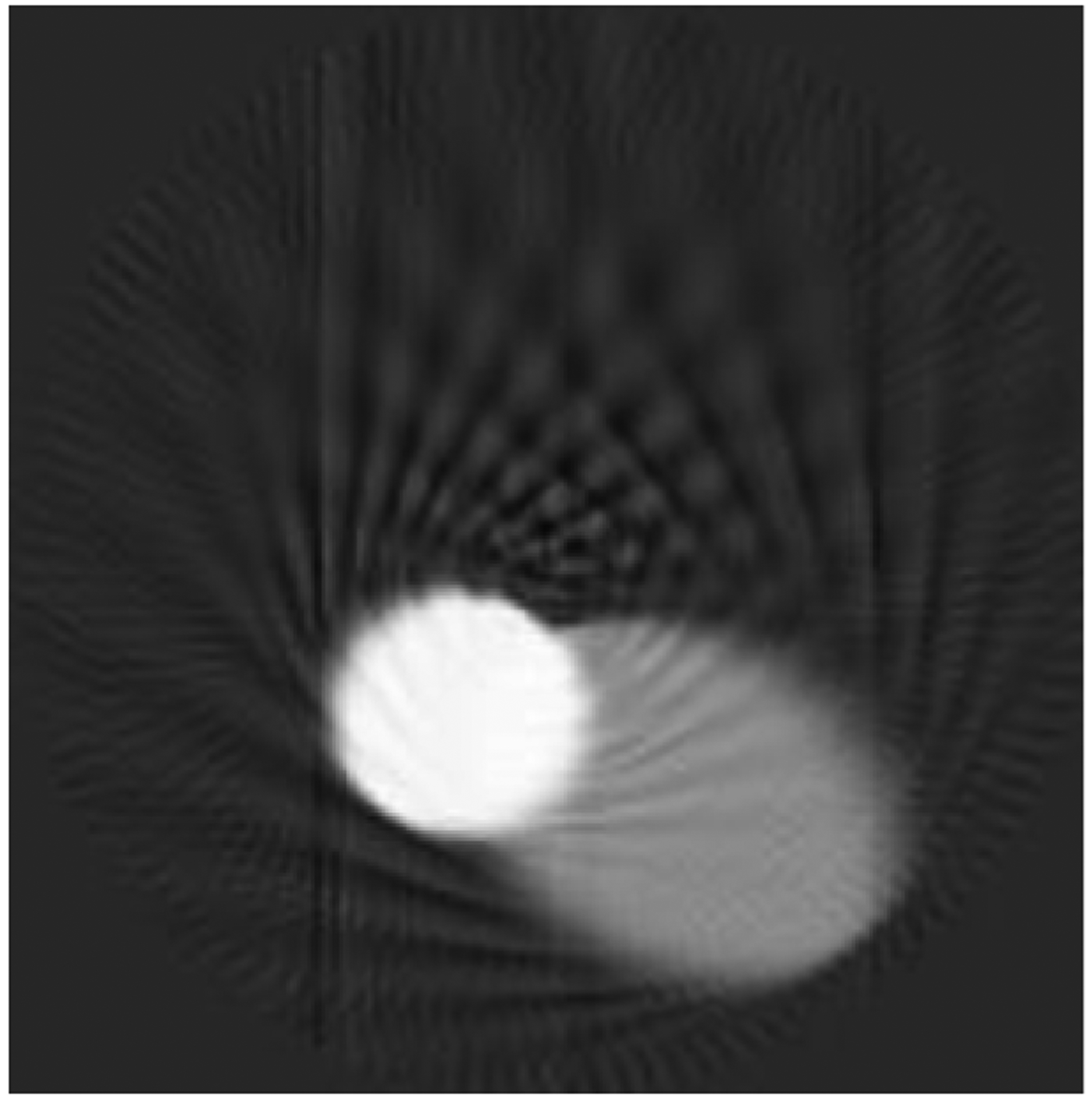
Reconstruction from linear-interpolated sinogram of elliptical phantom.

**Figure 11:**
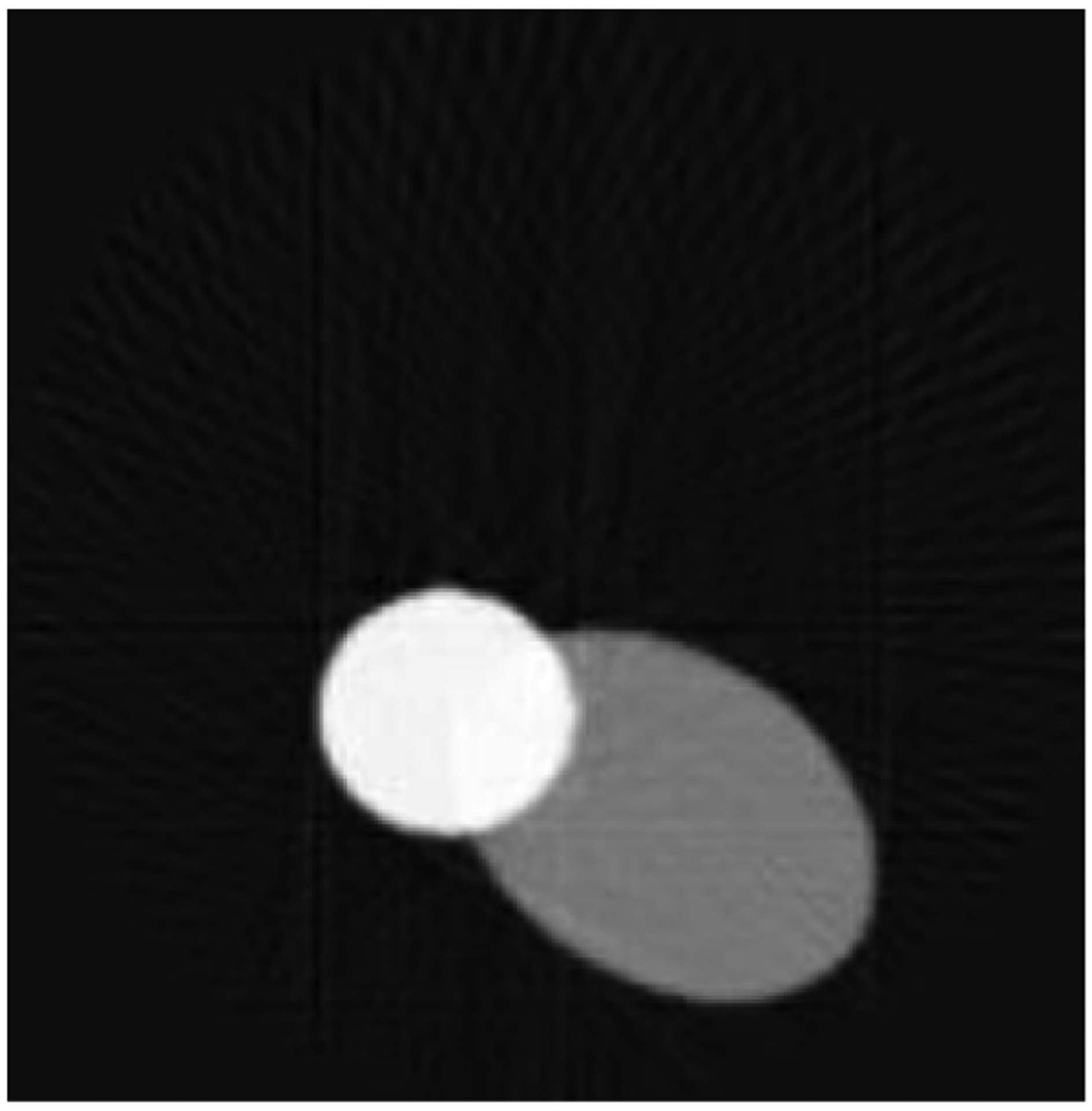
Reconstruction from model-interpolated sinogram of elliptical phantom.

**Figure 12:**
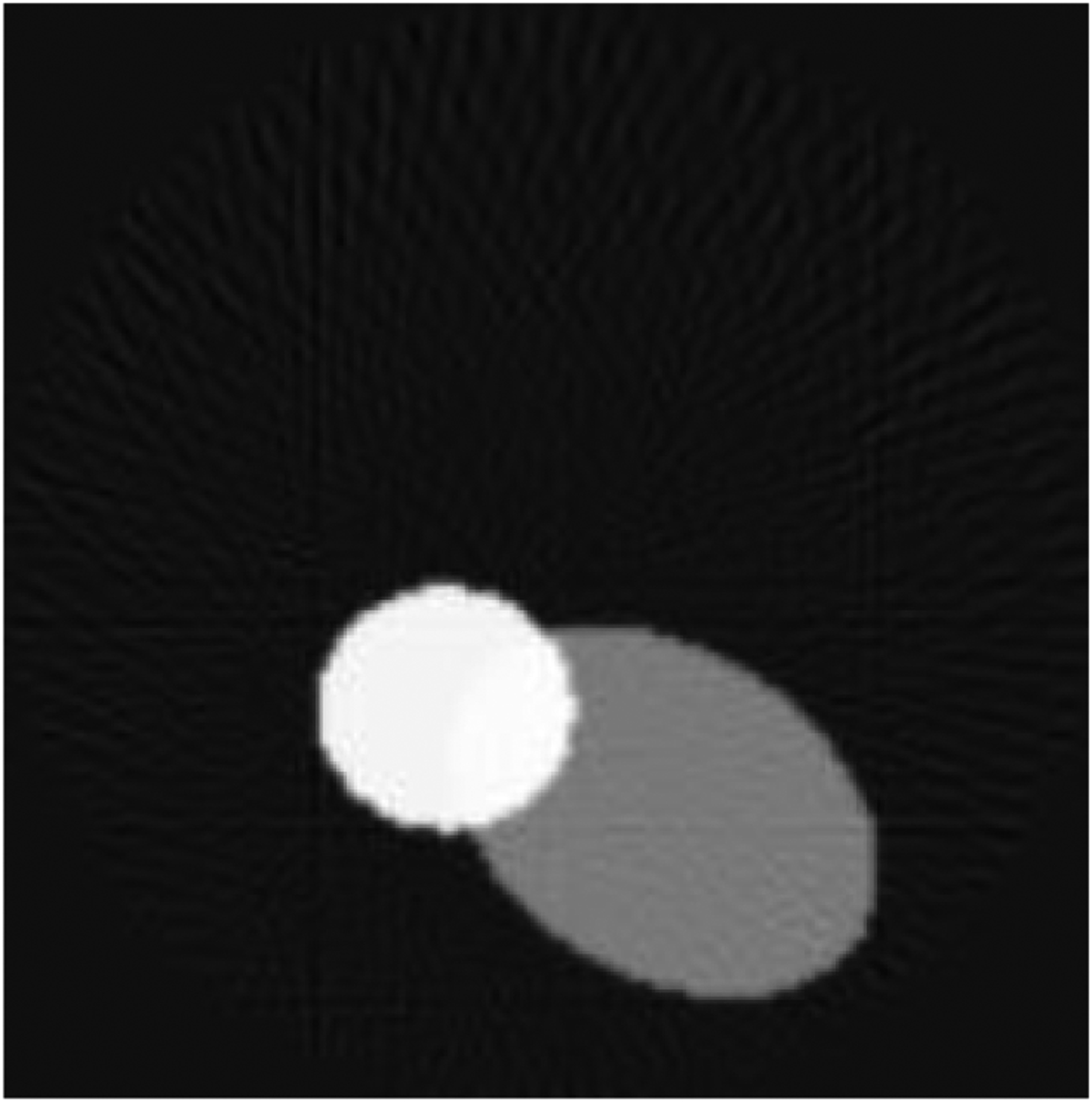
Reconstruction from full sinogram of elliptical phantom.

The computer simulated elliptical phantom studies are shown in Figures 9–12. Figure 9 shows the sparse view reconstruction. Figure 10 shows the sparse view reconstruction using the liner interpolation method to extend the sparse view into full view. Figure 11 shows the sparse view reconstruction using the proposed method to extend the sparse view into full view. Figure 12 shows the full view reconstruction with all measured data. The full view data consists of 128 views and the sparse view data consists of 16 views.

On test data, the average *mean-squared error* (MSE) differences between reconstructions and full reconstruction for simple ellipses phantoms are:

- 3.67 for model-predicted sinogram reconstruction.
- 17.70 for sparse sinogram reconstruction.
- 13.92 for linear image-resize sinogram reconstructions.

The model-reconstruction error is twenty-one percent of the sparse-reconstruction error. By comparison, the linear-reconstruction error is seventy-nine percent of the sparse-reconstruction error. The model results are superior to those from using linear interpolation.

**Figure 13:**
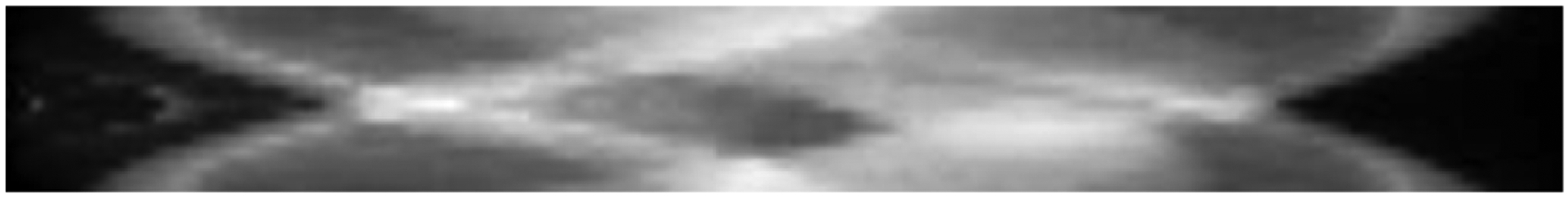
Sparse sinogram of downsized clinical image: 16 views, 128 collectors.

**Figure 14:**
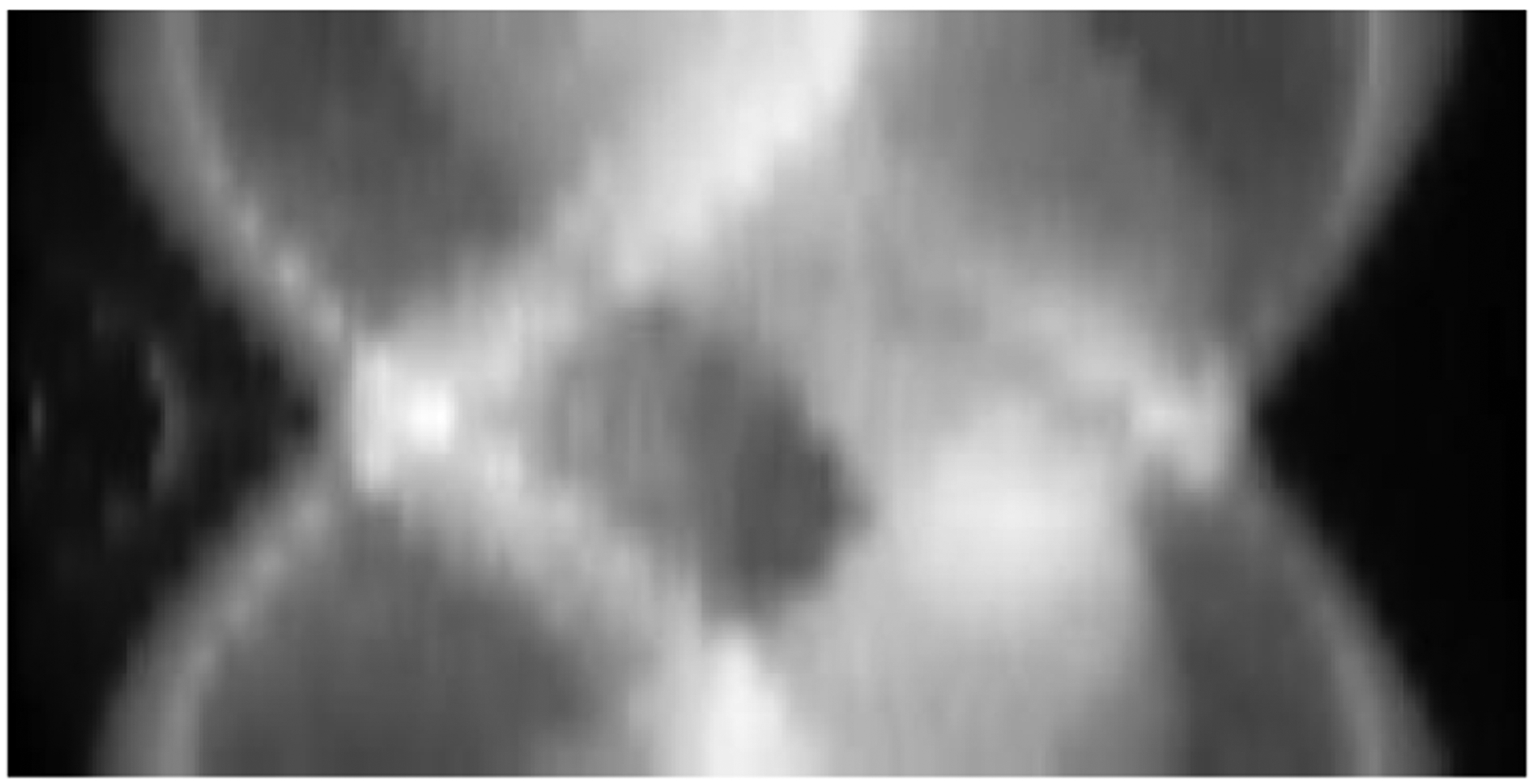
Linear-resized sinogram of downsized clinical image: 64 views, 128 collectors.

**Figure 15:**
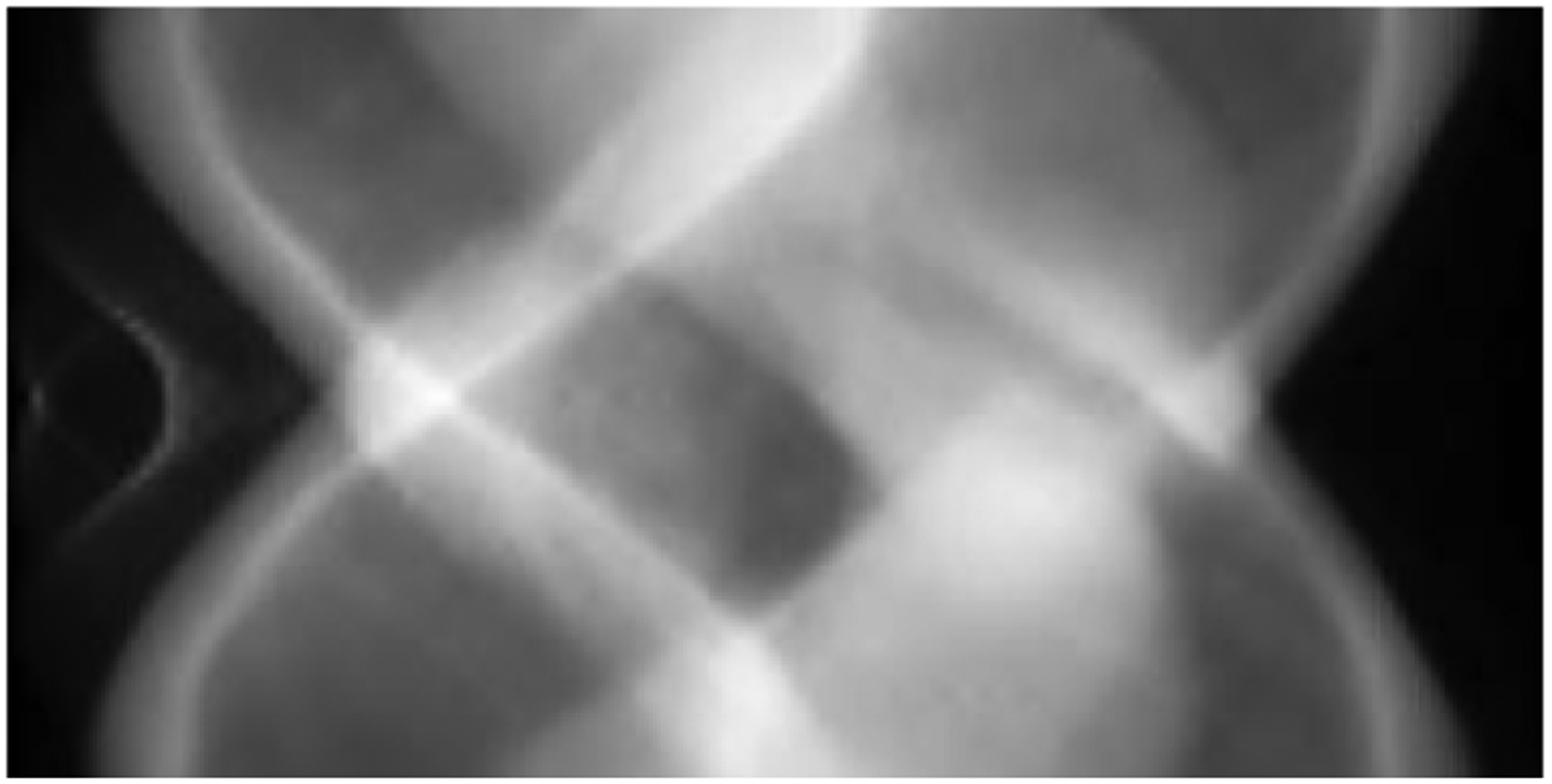
Model-interpolated sinogram of downsized clinical image: 64 views, 128 collectors.

**Figure 16:**
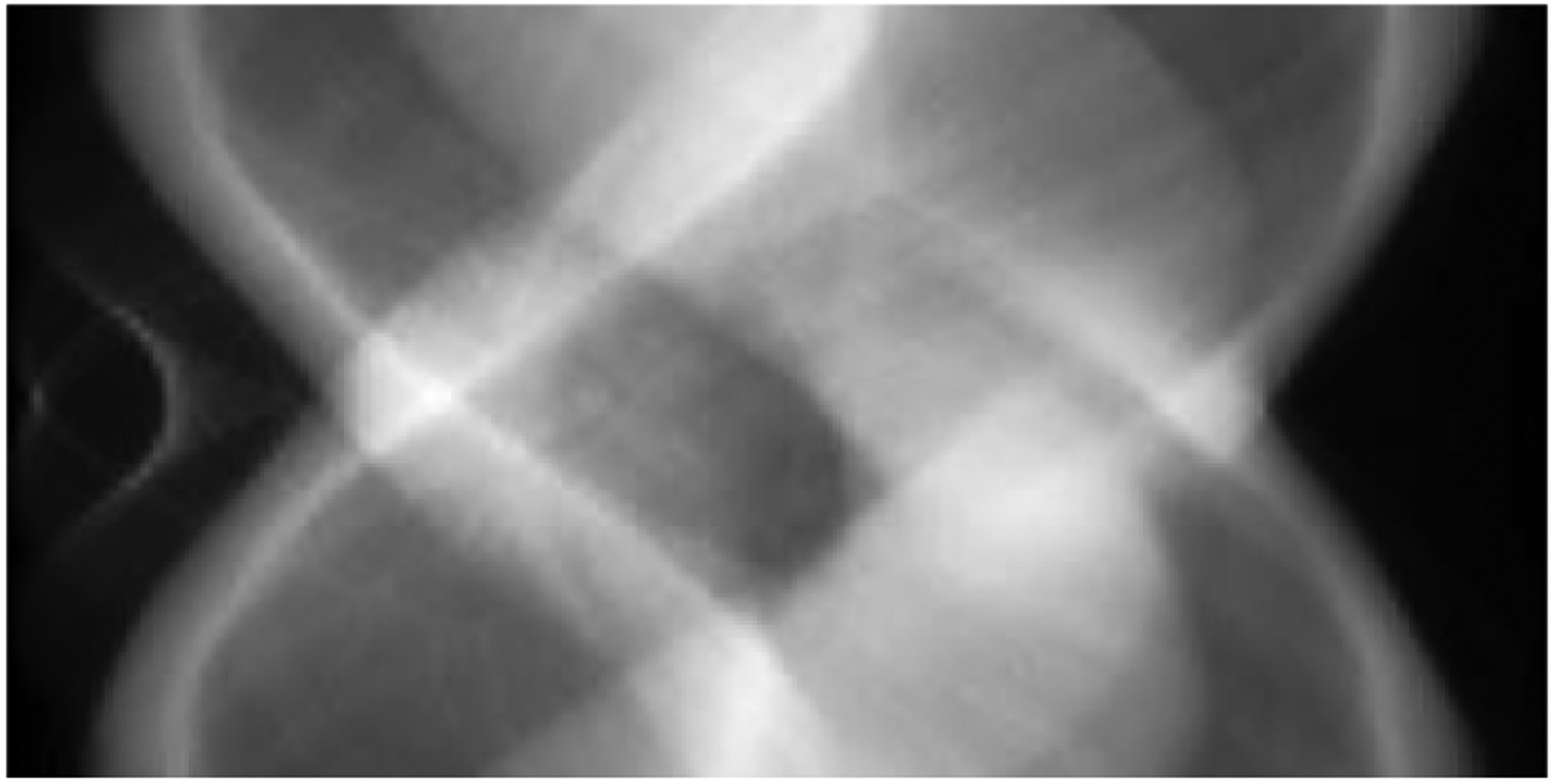
Full (ideal) sinogram of downsized clinical image: 64 views, 128 collectors.

**Figure 17:**
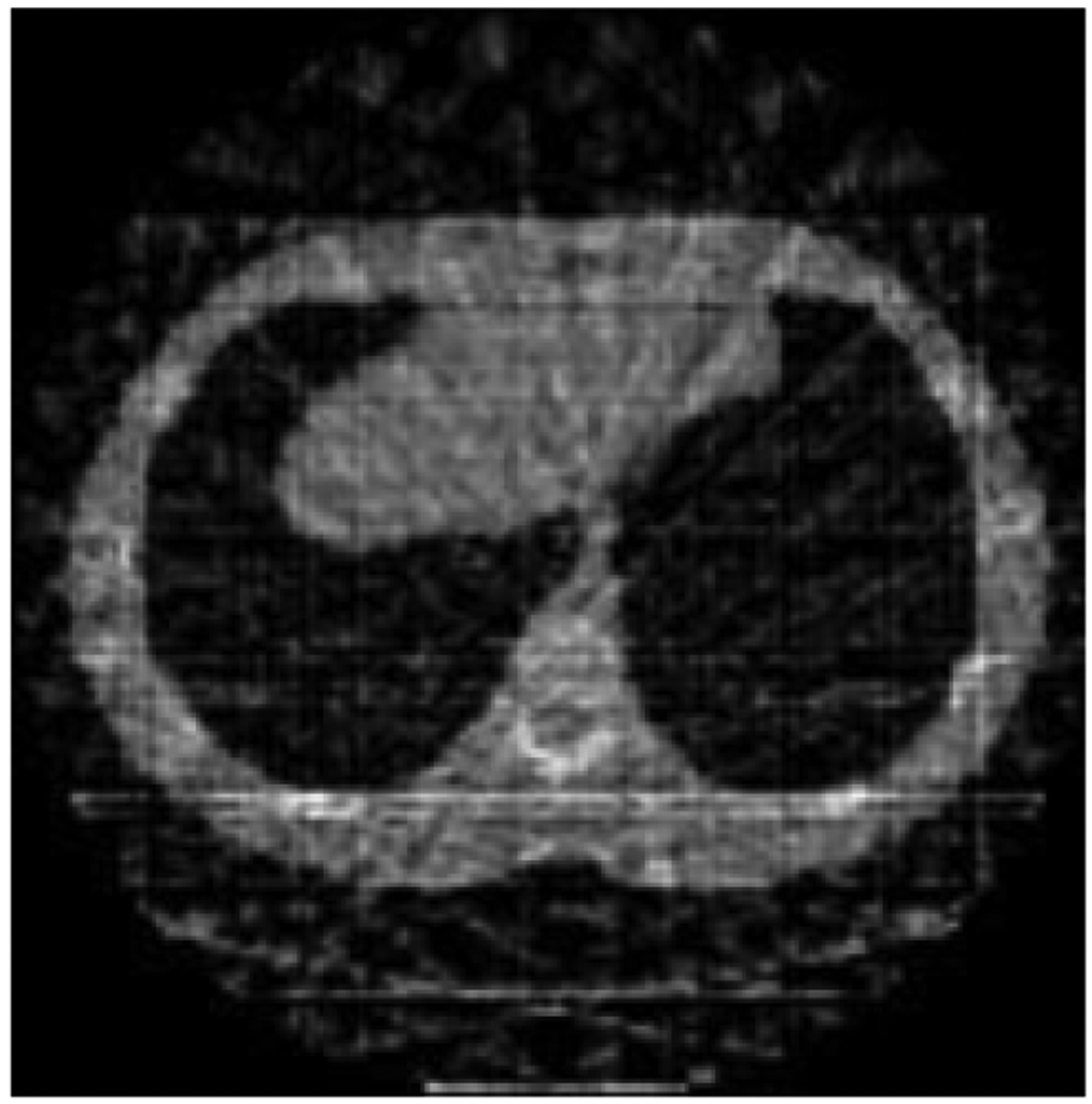
Reconstruction from sparse sonogram.

**Figure 18:**
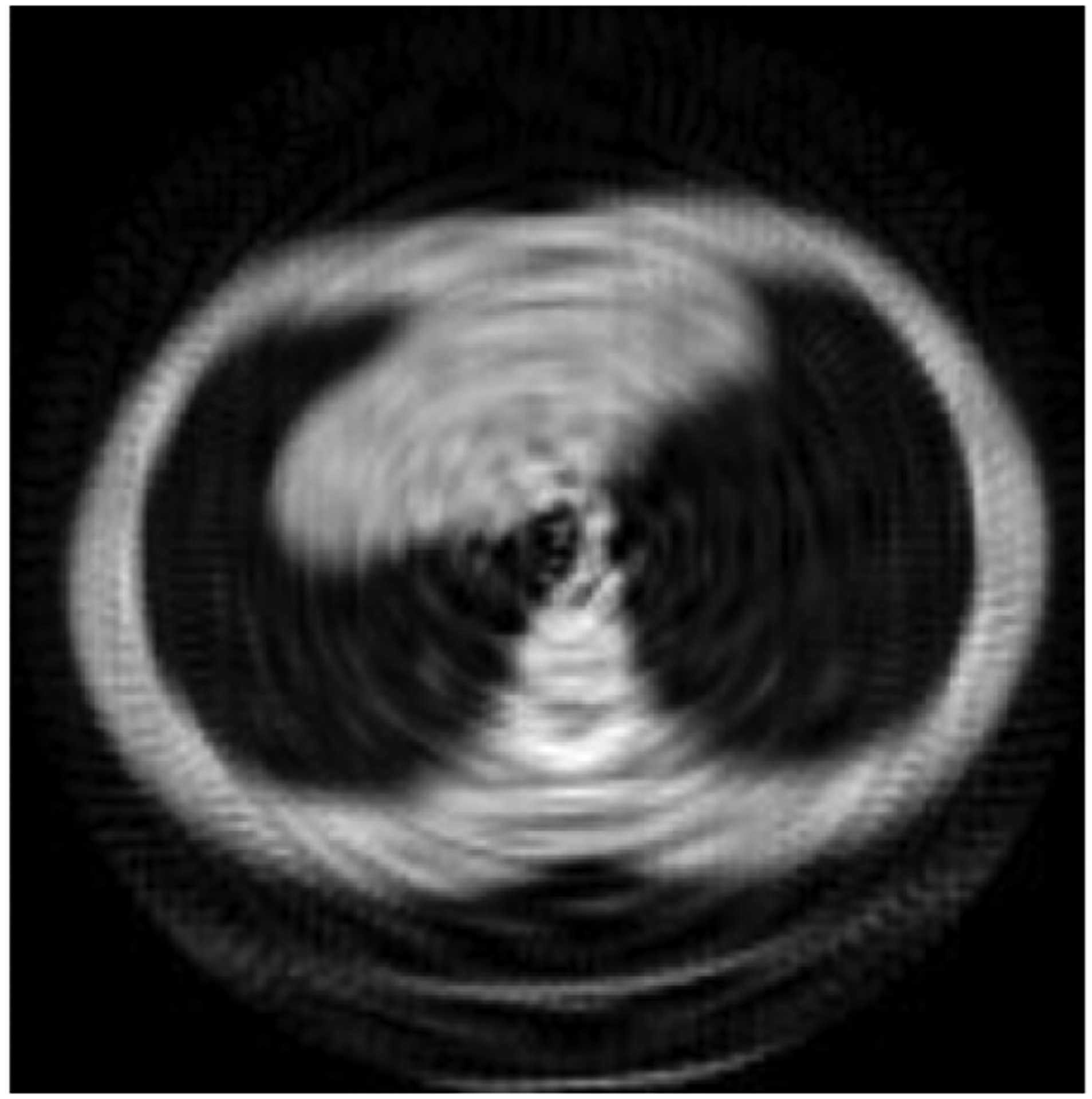
Reconstruction from linear-interpolated sonogram.

**Figure 19:**
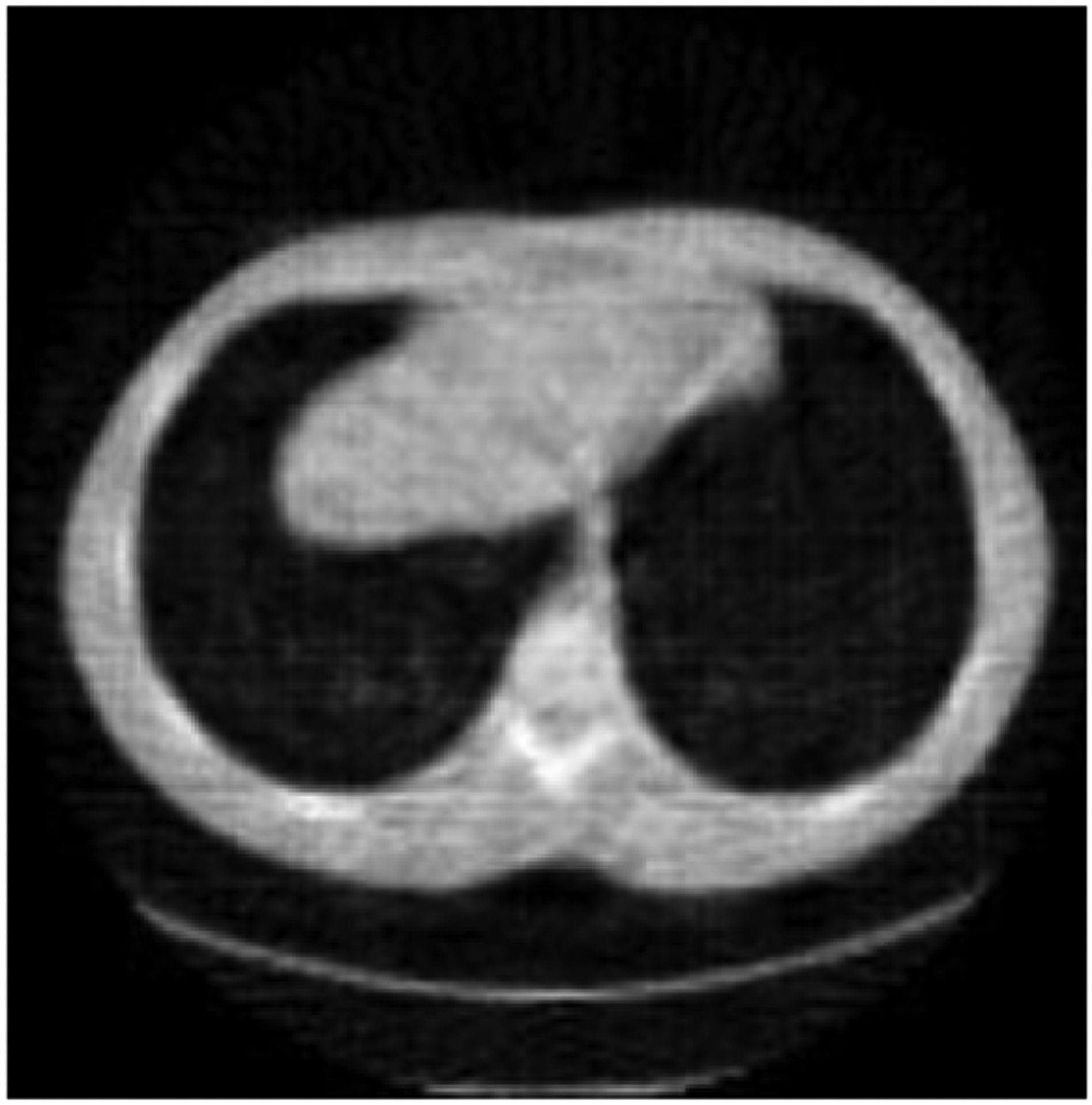
Reconstruction from model-interpolated sonogram.

**Figure 20:**
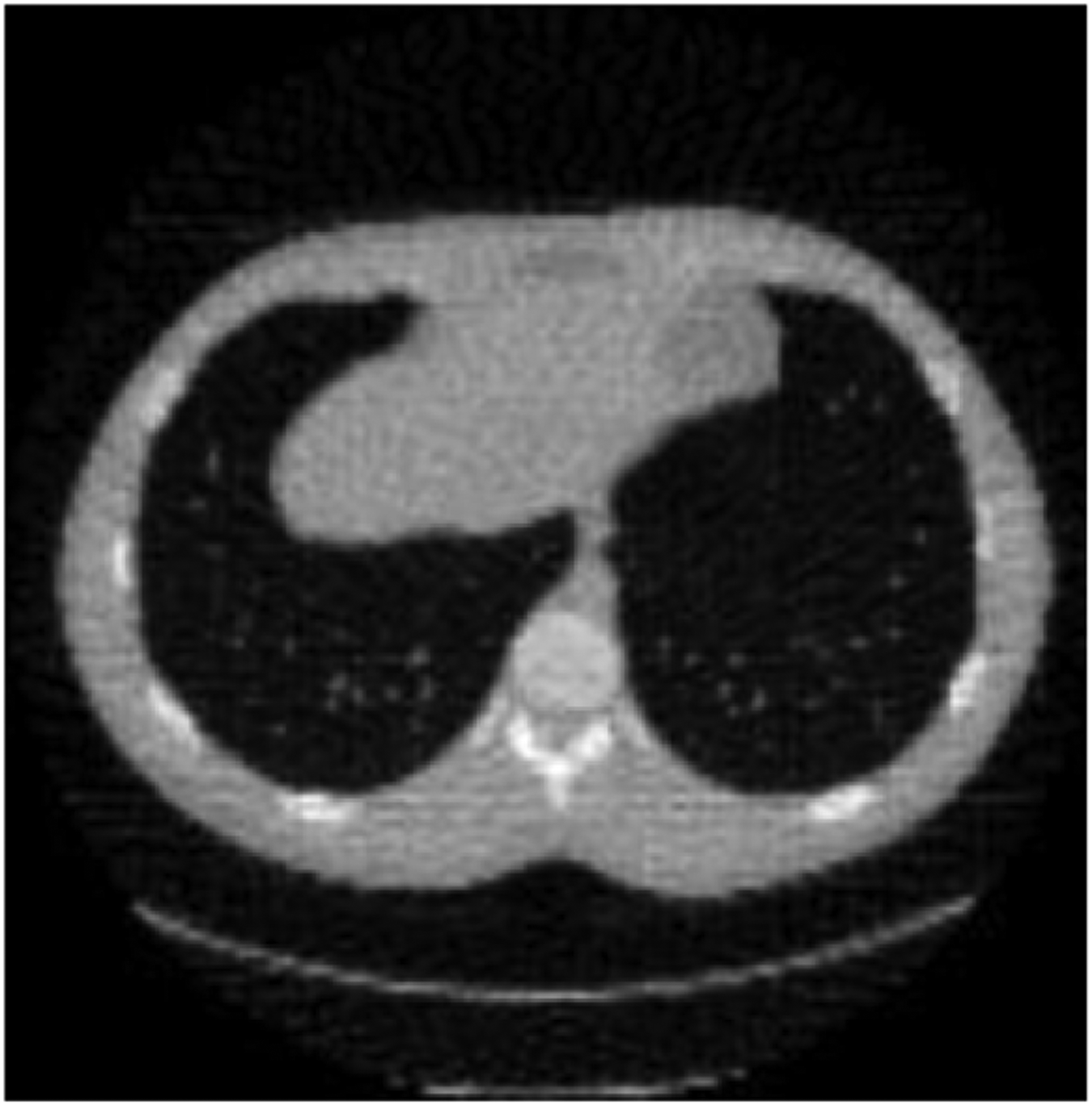
Reconstruction from full sonogram.

Results from the clinically measured data are shown in Figures 13–20. When the model is trained on more complex clinical phantoms, Figure 17 is the reconstruction of a sparse sinogram. Artifacts are visible as accordion-like gray lines in the image. The reconstruction of a linear-interpolated sonogram (Figure 18) demonstrates rotational tendencies. The reconstruction of the model-interpolated sinogram (Figure 19) shows a decrease in artifacts. The most ideal, full sinogram reconstruction is shown in Figure 20. The associated sinogram-domain data is displayed in Figures 13–16.

On clinical test data, the average *mean-squared error* (MSE) differences between reconstructions and full reconstruction are:

- 60.80 for model-predicted sinogram reconstruction.
- 152.56 for sparse sinogram reconstruction.
- 126.67 for linear image-resize sinogram reconstructions.

The model-reconstruction error is forty percent of the sparse-reconstruction error. By comparison, the linear-reconstruction error is eighty-three percent of the sparse reconstruction error. The model results are superior to those from using linear interpolation.

The error for the model-predicted reconstructions is less than error for the sparse sinogram reconstructions. The error for the model-predicated reconstructions is also less than error for reconstructions from linear-resized sinograms. The model can be declared a relative success. Other sparsities and views with 128 collectors. Using the same downsized clinical images as phantoms, similar models are trained and tested. Only the number of views and the sparsity (ratio between sparse views and full views) is changed. MSE values are recorded for reconstructions of test phantoms, using the full reconstruction as truth. Results are shown in Table 1.

## Conclusions

A deep-learning model based on SISR can be trained to interpolate a full sinogram from a sparse sinogram, reducing the error of the reconstructed image. A successful implementation of this model in a clinical setting will decrease patient radiation exposure and reduce associated health consequences, while still providing a high quality reconstructed image.

This model has room for improvement. Reconstructions from outputs of this model appear a little blurred when compared with reconstructions from a full sinogram. Adding a second part of the model that works in the reconstruction domain, as in “High quality imaging from sparsely sampled computed tomography data with deep learning and wavelet transform in various domains” [5], may improve the reconstruction of this image. This model should also be tested on noisy sinograms. In the presence of noise, the first layer of the model may benefit from a larger kernel size.

## Figures and Tables

**Table 1:** MSE for models of varying views and sparsities.

Sparse views	Full Views	Model MSE as fraction of sparse MSE	Linear MSE as fraction of sparse MSE
128	256	0.70	1.76
85	256	0.61	2.62
64	256	0.51	1.40
64	128	0.56	1.20
43	129	0.47	1.13
32	128	0.41	1.01
32	64	0.49	0.98
21	63	0.44	0.93
16	64	0.40	0.83

## Data Availability

The data that support the findings of this study are available from The Cancer Imaging Archive but restrictions apply to the availability of these data, which were used under license for the current study, and so are not publicly available. Data are however available from the authors upon reasonable request and with permission of The Cancer Imaging Archive.

## Abbreviations

CT: Computed tomography
FBP: Filtered backprojection
SISR: Single image super-resolution
